# Baseline epigenetic clock measures in the Northern Ireland Cohort for the Longitudinal Study of Ageing (NICOLA)

**DOI:** 10.1186/s13104-026-07771-0

**Published:** 2026-03-19

**Authors:** Claire Hill, Claire Potter, Laura Smyth, Jill Kilner, Angela Scott, Charlotte Neville, Frank Kee, Bernadette McGuinness, Amy Jayne McKnight

**Affiliations:** Centre for Public Health, Queen’s University Belfast, Belfast, UK

**Keywords:** Epigenetic, Methylation, EPIC, Epigenetic clock, Ageing, NICOLA, Association

## Abstract

**Objectives:**

DNA methylation (DNAm) can change dynamically in response to intrinsic or external exposures. Characteristic methylation profiles are associated with accelerated biological ageing and poorer health outcomes. CpG methylation levels for individuals within the Northern Ireland Cohort for the Longitudinal Study of Ageing (NICOLA) were previously generated from baseline blood-derived DNA typed on the Infinium MethylationEPIC array. Harnessing this methylation data, epigenetic clock measures were determined, adjusting for chronological age and white blood cell count (CD8T, CD4T, Natural killer, B-cell, Monocytes and Granulocytes).

**Data description:**

The NICOLA study was designed to be representative of the Northern Ireland population at Wave 1 and involved the recruitment of 8,283 people from across Northern Ireland. Participants over the age of 50 were recruited. Other household members (spouses/partners) were allowed to take part, even if they were under the age of 50. 2.3% of participants were under the age of 50. Around 50% of participants were aged between 50 and 65, and 10% of participants were over the age of 80 years. This dataset includes epigenetic clock measures for a subset of 1,870 (48% male) participants within the NICOLA cohort, with a mean age of 64.1 ± 9.37 years. The following 10 epigenetic clocks were generated: (± PC)PhenoAge, (± PC)GrimAge, (± PC)Horvath1, (± PC)Hannum, PCDNAmTL and DunedinPACE. ±PC represents the presence or absence of principal component adjustment. This document describes NICOLA’s epigenetic clock dataset, which is ~ 2.5Gb in size.

## Objective

The NICOLA study involved recruiting 8283 community-dwelling adults aged 50 and older from a randomly selected list of Northern Ireland addresses. The study included an additional 195 spouses or partners, with spouses/partners included even if under the age of 50. The gender distribution at baseline was 45% male. The study investigates how ageing affects, and is affected by, factors such as health, lifestyle, environmental, and social circumstances. Participants underwent thorough evaluations, including computer-assisted interviews, self-administered questionnaires, physical measurements, and the collection of biological samples.

Blood-derived DNA was extracted from buffy coats available for a subset of NICOLA participants who attended the health assessment [1–3]. Using blood derived DNA, methylation at 862,927 CpG sites with single site resolution was determined in a sub-set of participants using the Illumina EPIC array [4]. The data described in this data note is part of the wider NICOLA cohort, a study led by the Centre for Public Health at Queen’s University Belfast (https://www.qub.ac.uk/sites/NICOLA/). Descriptions of the NICOLA cohort, as well as details DNA extraction and Illumina EPIC array protocols used to generate the methylation data are described in the 2017 Early Findings Report [5], cohort profile [6], and the 2021 Health Assessment Report [4]. The NICOLA study website contains details of all the data that is available through a fully searchable data dictionary (https://www.qub.ac.uk/sites/NICOLA/ForResearchers/).

Harnessing the epigenetic data, a range of epigenetic clock variables were derived. Epigenetic clocks are used as a measure of ageing [7]. These clocks were selected to span 1st (Horvath [8] and Hannum [9]), 2nd (PhenoAge [10] GrimAge [11] DNAmTL [12]) and 3rd generation epigenetic clocks (DunedinPACE [13]). Epigenetic clocks can be used to determine accelerated ageing, by comparing chronological age with epigenetic age. Derived epigenetic clock measures for 1,870 NICOLA participants are available via the European Genome-phenome Archive (EGA) (EGAD50000002063).

### Data description

The NICOLA study aims to pinpoint risk factors associated with age-related diseases, to gather evidence to inform policies and interventions that foster healthy ageing. Results can be compared with similar cohorts around the world, helping to advance healthy ageing on a global scale. Wave 1 was completed in 2020/21 [4], Wave 2 completed in 2023, and Wave 3 is currently ongoing.

A subset of 3,741 NICOLA participants attended the Wave 1 health assessment [1–3]. Biological samples were available for 3,514 participants who attended the health assessment [4]. DNA was derived from venous blood samples collected during this health assessment. Blood sample handling and DNA extraction are described previously [4]. Funding was available for 2,000 samples to undergo DNA methylation profiling. The first 2000 samples that had sufficient, high-quality DNA extracted from venous blood samples were submitted for methylation typing on the EPIC array [4]. 8 samples had insufficient DNA and 16 withdrew consent [3], with 1,976 samples remaining.

Samples were randomised across plates for bisulfite treatment and Illumina Infinium Methylation EPIC 1.0 array analysis with 8 blind duplicates; analysis of blind duplicates revealed > 97% concordance for all CpG sites.

#### Quality control

The Illumina EPIC array data underwent quality control (QC) processing with a specific focus on epigenetic clock generation. R version 4.3.0 was used [14], with the libraries installed for the QC processing detailed in Supplementary Table 3.

- Log2 Median Unmethylated Intensity (MUI) and Log2 Median Methylated Intensity (MMI) were determined. 9 samples were removed as they had log2 MUI and log2 MMU less than 10.
- Illumina has 17 control metrics to check sample quality via the ewastools package[15]. In total, 50 samples failed at least one Illumina control metric and were excluded.
- Sex was predicted using check_sex (ewastools), correcting for dye-bias (correct_dye_bias function). 31 samples displayed a sex mismatch (where predicted sex did not match reported sex) (Y less than 0.3 (male mismatch) or (X less than 0.75 (female mismatch)).
- Visual inspection of sex clustering plot was carried out to remove non-clustering samples (X > 0.95 & Y > 0.85), with less than 5 samples identified.
- Sample relatedness was determined using single nucleotide polymorphism (SNP) probes with an rsID label (correct_dye_bias, mask = 0.01, without normalization, check_snp_agreement). 47 related pairs were identified, with one sample with each related sample pair was retained (preferentially excluding samples that were already highlighted as exclusions in preceding QC steps).

In total, 1870 (106 removed) samples remained for downstream analysis.

The following probe quality control was carried out:

- Data was normalised using the ‘*noob*’ method.
- All values with a detection p-value of > 0.01 and/or a beadcount of under 4 were labeled as unreliable. All probes with over 5% unreliable values were cut. 35,353 probes were removed in this step.

In total, 830,738 probes remained for downstream analysis.

The epigenetic clock subset is 48% male, with a mean age of 64.1 ± 9.37 (Supplementary Tables 1 and 2). All supplementary figures and tables are available at the Open Science Framework: 10.17605/OSF.IO/ZD37J (NICOLA Data Products – Associated files – Baseline epigenetic clocks) [16].

Supplementary Tables 4–7 summarises the epigenetic clocks available. Extended methods are included in the supplementary. Protocols were harmonsied between NICOLA, The Irish Longitudinal Study on Ageing (TILDA) and the Health and Retirement Study (HRS), enabling cross-country analyses [17].

Plots highlighting the correlation of (± PC)PhenoAge, (± PC)GrimAge, PCDNAmTL, (± PC)Horvath1, (± PC)Hannum and PACE clocks with chronological age are shown in Supplementary Figs. 2–7. Correlation values stratified by sex are shown in Supplementary Table 7.

**Table 1:** Overview of data files/data sets

Label	Name of data file/data set	File types (file extension)	Data repository and identifier (DOI or accession number)
Data set 1	EGAF00008971437	.csv	EGA ID:EGAD50000002063 (https://identifiers.org/ega:EGAD50000002063) [18]
Data file 1	Baseline epigenetic clocks/EpigeneticClocks_NICOLA_datanote_supplementary_090126.pdf	.pdf	Open Science Framework (10.17605/OSF.IO/ZD37J) [16]

### Limitations

Clearly state the limitations of the data. This could be any shortcoming that occurred during data collection, small sample sizes (which prevented the data to be used as part of a research paper) or outdated data among others. We believe all sound data is valuable and deserves publication. Honest and transparent description of any limitations will not affect editorial assessment. Limitations may be presented in bullet point format.

- Epigenetic clocks have only been determined for a sub-set (*N* = 1,870) of the larger NICOLA cohort; this is the maximum number available after quality control within the DNA methylation subset (*N* = 1,976) and is representative of the larger NICOLA cohort [3].
- At present, only cross-sectional epigenetic data is available; future repeated measures and samples will be collected to facilitate longitudinal assessment [19]. Wave 2 follow-up data (Computer-Assisted Personal Interviews and self-completed questionnaire) is available, with Wave 3 recruitment including a health assessment, which is currently ongoing at the time of publication.
- A selection of established epigenetic clocks have been generated for this release, with scope to calculate additional epigenetic clocks. The raw data to do so is available upon request to the NICOLA data access committee (https://www.qub.ac.uk/sites/NICOLA/InformationforResearchers/).

## Data Availability

The data described in this data note can be freely accessed via the European Genome-phenome Archive (EGA) (EGA ID: EGAD50000002063) . Please see Table 1 for details and links to the data. To access this data from the EGA you will need to provide information about your research project and intended use of the data. Registered EGA users can click on “Request access” on the EGA EGAD50000002063 dataset page to submit an access request. The EGA facilitates the secure distribution under controlled access of personally identifiable genetic and phenotypic data. The NICOLA Data Access Committee (DAC) (EGA ID: EGAC00001003504) will receive this request and are responsible for reviewing and approving requests, and for data release to external requestors.

## Abbreviations

NICOLA: Northern Ireland Cohort for the Longitudinal Study of Ageing
MUI: Median Unmethylated Intensity
MMI: Median Methylated Intensity
PC: Principal component
IEAA: Intrinsic epigenetic age acceleration
EEAA: Extrinsic epigenetic age acceleration
